# “Omics” Signatures in Peripheral Monocytes from Women with Low BMD Condition

**DOI:** 10.1155/2018/8726456

**Published:** 2018-03-18

**Authors:** Bhavna Daswani, M. Ikram Khatkhatay

**Affiliations:** Division of Molecular Immunodiagnostics, National Institute for Research in Reproductive Health, ICMR, J. M. Street, Parel, Mumbai 400012, India

## Abstract

Postmenopausal osteoporosis (PMO) is a result of increased bone resorption compared to formation. Osteoclasts are responsible for bone resorption, which are derived from circulating monocytes that undertake a journey from the blood to the bone for the process of osteoclastogenesis. In recent times, the use of high throughput technologies to explore monocytes from women with low versus high bone density has led to the identification of candidate molecules that may be deregulated in PMO. This review provides a list of molecules in monocytes relevant to bone density which have been identified by “omics” studies in the last decade or so. The molecules in monocytes that are deregulated in low BMD condition may contribute to processes such as monocyte survival, migration/chemotaxis, adhesion, transendothelial migration, and differentiation into the osteoclast lineage. Each of these processes may be crucial to the overall route of osteoclastogenesis and an increase in any/all of these processes can lead to increased bone resorption and subsequently low bone density. Whether these molecules are indeed the cause or effect is an arena currently unexplored.

## 1. Introduction

Postmenopausal osteoporosis (PMO) is characterized by low bone mineral density (BMD) and microarchitectural deterioration of bone tissue, wherein the genesis and activity of osteoclasts (bone resorbing cells) supersede those of osteoblasts (bone forming cells); thus, bone resorption exceeds bone formation [1, 2]. The outcome of PMO is increased susceptibility to fragility fractures which can lead to substantial morbidity and mortality. Even though the primary underlying cause is increased bone resorption compared to formation, the pathological mechanism of PMO is complex and multifaceted, involving many factors such as genetics, levels of sex hormones and cytokines, lifestyle, and environmental factors. Of late, the role of immune cells and their complex interplay in bone biology have also been recognized, which has led to the advent of the term “osteoimmunology” [3]. Amongst these immune cells, monocytes have gained considerable importance, not only because they secrete important cytokines that modulate bone remodeling, but also because they are precursors of osteoclasts and they travel from the blood towards the bone to differentiate into osteoclasts. Thus, monocytes can provide insights into the initial stages of osteoclastogenesis. In addition, monocytes' easy accessibility (in circulation) compared to osteoclasts (that reside deep within bone cavities) has encouraged researchers to utilize the former to unravel the complex etiology of PMO. In fact, a recent study proposed the utilization of monocytes for screening and monitoring of PMO [4].

Further, it has been shown that the number of monocytes is not significantly different between osteoporotic and nonosteoporotic women; however, the ex vivo osteoclastogenic potential is significantly higher in monocytes derived from osteoporotic women [5]. Also, monocytes derived from postmenopausal women have higher osteoclastogenic potential than those derived from premenopausal women [6]. Since osteoporosis is known to have a strong genetic component, intrinsic differences in monocyte gene expression patterns can contribute to extrinsic phenotypes. Therefore, in the last decade or so, researchers have studied monocytes from women with low versus high BMD using comparative “omics” methods, and this has resulted in a large surge of candidate molecules that may be implicated in the pathophysiology of PMO. The usefulness of monocytes in overall bone research has been reviewed previously [7]. The present review addresses all the studies on monocyte “omics” in relation to BMD while also providing the outcome of these studies in the form of a list of candidate molecules.

## 2. Monocyte's Journey from Blood to Bone to Become Osteoclasts

Monocytes circulate in the blood for 1 to 3 days, during which they enter various tissues and can differentiate into osteoclasts or resident macrophages (like splenic macrophages, kupffer cells in the liver, and alveolar macrophages in the lungs), depending upon the tissue microenvironment. Apart from M-CSF (macrophage colony stimulating factor) which provides survival signals, commitment to the osteoclast lineage also requires the presence of RANKL (receptor activator of NFkB ligand), a crucial osteoclast differentiation factor [8]. Although it was known that osteoclasts have a hematopoietic origin, it was only in the last few decades that compelling evidence was provided for the fact that circulating monocytes are precursors of osteoclasts. In 1981, it was shown that when radiolabeled monocytes were injected into syngenic mice, the femurs of recipient mice showed labeled osteoclasts [9]. Several studies in the following years showed that osteoclast precursors lie in the monocyte fraction and these precursors show characteristic features of monocytes (CD14 positive cells) [8, 10–13]. It also became clear that monocytes express RANK and c-fms (CD115), and the presence of their ligands RANKL and M-CSF, respectively, was necessary and sufficient for osteoclastogenesis to occur [8, 14, 15]. In a recent study in mice, fluorescence-based methods were used* in vivo* to confirm that osteoclasts can be generated from circulating monocytes. and the authors suggested that this detour of monocytes from bone marrow to blood to bone is possibly because bone resorption does not occur homogeneously in all bone tissues [16]. Further, monocytes are known to have two subsets, CD16 positive and CD16 negative, the latter being classical monocytes constituting around 90 to 95% of total monocytes. Notably, it was also found that osteoclasts originate from the CD16 negative subset of monocytes and this has been experimentally proven [17–19], despite certain arthritic conditions which may be exceptions to this rule.

The journey of a peripheral monocyte to osteoclast involves three early stages: chemotaxis towards the site for osteoclast formation, adhesion to the endothelial cells near the bone, and finally transendothelial migration into the bone milieu. In this context, soluble RANKL can stimulate chemotaxis of human monocytes via its receptor RANK [20, 21]. This explains the migration of monocytes towards the bone microenvironment wherein soluble RANKL is present in high amounts. Also, under the influence of RANKL or parathyroid hormone, osteoblasts secrete MCP-1 (monocyte chemoattractant protein-1), a potent monocyte recruitment factor that attracts monocytes via C-C chemokine receptor type 2 (CCR2) [22, 23]. Also, chemokine (C-X-C motif) receptor 4 (CXCR4) on monocytes responds to stromal cell-derived factor-1 (SDF-1) secreted by bone vascular endothelial and marrow stromal cells [24, 25], and CX3CR1 on monocytes responds to CX3CL1 secreted by osteoblasts [26]. One of the most important breakthroughs in this field was the discovery of the role of sphingosine-1 phosphate (S1P) which acts via its receptors S1PR1 and S1PR2 present on monocytes, as a vital regulator of monocyte bone homing in mice. The investigators showed that S1PR1 promotes chemotactic movements into blood circulation (where S1P is high), whereas S1PR2 has chemorepulsive properties and promotes movement into the bone microenvironment [27, 28]. These findings have also shed new light on the bone protective effect of vitamin D_3_* in vivo *as they found that vitamin D_3_ controls monocyte migration towards the bone microenvironment by reducing the expression of S1PR2 both* in vitro* and* in vivo* [27–29]. Taken together, monocyte migration to the bone milieu is crucial to osteoclastogenesis and is regulated by many molecules which probably work in concert; hence, deciphering the entire molecular repertoire is still the subject of current research. Most of the above-mentioned studies are in mice; fortunately, the “omics” era opened a window of opportunity to simultaneously explore a plethora of molecules in monocytes between low and high bone density conditions. Accordingly, many studies have explored monocyte gene expression in relation to BMD using RNA and protein profiling methods described in the next sections. In addition, few studies have used data from these “omics” studies and applied bioinformatic tools to identify relevant molecular pathways and networks pertinent to PMO. A list of differentially expressed molecules in monocytes in low versus high BMD found using “omics” methods along with experimental validation is provided in Table 1.

## 3. Monocyte mRNA Profiling in Low versus High BMD Condition

More than a decade ago, Deng and colleagues carried out an elegant study to explore monocyte transcriptome in relation to osteoporosis. They applied the microarray technique to explore monocyte mRNA derived from premenopausal and postmenopausal Caucasian women with low versus high BMD condition [30]. They found that chemokine receptor 3 (CCR3), glucocorticoid receptor (GCR), and histidine decarboxylase (HDC) were upregulated in low BMD condition which was confirmed by quantitative reverse transcriptase-polymerase chain reaction (qRT-PCR) [30]. These genes were related to monocyte chemotaxis, sensitivity to glucocorticoids (steroids used for treatment of secondary osteoporosis), and histamine production, respectively [30]. Another study was published on the same lines which involved premenopausal Chinese women and three genes were upregulated in low BMD condition, namely, signal transducer and activator of transcription 1 (STAT1), guanylate binding protein 1 (GBP1), and C-X-C motif chemokine 10 (CXCL10) [31]. The first two were confirmed by qRT-PCR and these genes were mainly associated with interferon signaling required for monocyte-to-osteoclast differentiation [31]. Subsequently, a study was conducted on monocyte gene expression profile of 168 genes which were relevant to BMD in both Chinese and Caucasian women. Of the 168 genes, 13 were found to be differentially expressed which were related to interferon signaling, of which STAT1 was significantly upregulated in low BMD condition [32]. This encouraged research examining single nucleotide polymorphisms (SNPs) of STAT1 gene in association with BMD, and the same authors found that two SNPs (rs2030171 and rs10199181) showed significant association with BMD in Caucasians [32]. In the following year, a bootstrap-based regression method was applied to identify altered gene expression in monocytes using previous microarray data wherein genes were segregated as BMD related and menopause related [33]. They found that the former category included genes related to cellular protein metabolism and the latter were upstream or downstream targets of estrogen receptors [33]. Subsequently, pathway analyses using graph clustering method were used to group differentially expressed genes from monocyte Affymetrix microarray datasets available online and two gene clusters were found to be enriched, namely, immune system and stimulus response [34]. Further, a recent study published in 2015 showed that apoptosis related genes were downregulated in monocytes in low BMD condition [35]. The investigators confirmed the differential expression of apoptosis induction gene, polo-like kinase 3 (PLK3), by qRT-PCR [35]. In addition, they performed a meta-analysis across 4 microarray datasets from their previous studies (including premenopausal and postmenopausal Caucasian and premenopausal Chinese females) and found genes related to induction of apoptosis, the majority of which were downregulated in low BMD condition. Of these genes, PLK3 and death domain-associated protein 6 (DAXX) were replicated across all four datasets [35]. Since circulating monocytes have two fates, either apoptosis or differentiation, they suggested that decreasing apoptosis is a mechanism for increasing monocyte survival and thus migration and differentiation into osteoclasts [35].

## 4. Monocyte Protein Profiling in Low versus High BMD Condition

Comparative proteomics of monocytes in relation to BMD was first reported in 2008 from premenopausal Chinese women with low versus high BMD condition, wherein two-dimensional electrophoresis was employed and 5 proteins were differentially abundant including Ras suppressor protein 1 (RSU1), gelsolin (GSN), and manganese containing superoxide dismutase (SOD2) which were upregulated, while glutathione peroxidase 1 (GPX1) and prolyl 4-hydroxylase b subunit (P4HB) were downregulated in low BMD condition [36]. The differential abundance was confirmed using western blot and these proteins were related to oxidative stress (SOD2 and GPX1), cell migration (GSN), adhesion (RSU1), and apoptosis regulation (P4HB) [36]. On the basis of these findings, SNPs in the SOD2 gene and its mRNA levels in monocytes from premenopausal Chinese women were later studied [37]. Three SNPs (rs7754103, rs7754295, and rs2053949) were significantly associated with BMD as well as with its mRNA levels [37]. In the following studies, label-free liquid chromatography-mass spectrometry (LC-MS/MS) was employed to compare monocyte proteins from women with low versus high BMD. Initially, in postmenopausal Caucasian women, Annexin A2 (ANXA2) was found to be upregulated in low BMD condition and this was validated at mRNA and protein levels in separate cohorts [38]. Also, SNP analysis of ANXA2 gene in unrelated adult Caucasians showed that three SNPs (rs7163836, rs11633619, and rs11633657) were significantly associated with BMD [38]. Moreover, recombinant Annexin A2 protein showed an increase in transendothelial migration of monocytes* in vitro,* besides its known function in osteoclast formation [38]. In a similar study, in premenopausal Caucasian women, using label-free LC-MS/MS, gelsolin (GSN) was downregulated in low BMD condition which was confirmed at mRNA and protein levels in two separate cohorts of Caucasian women [39]. This was then extended to SNP analysis of GSN gene which revealed that one SNP (rs767770) was associated with BMD in Caucasian females but not in Chinese females or males of either of the two ethnicities [39]. Recently, network based analyses were employed to analyze the proteomic data of monocytes from postmenopausal Caucasian women, generated by label-free LC-MS/MS [40]. In this study, it was found that peptidylprolyl isomerase A (PPIA), similar to peptidylprolyl isomerase A isoform 1 (LOC654188), transgelin 2 (TAGLN2), and isoform long of 14-3-3 protein beta/alpha (YWHAB) were downregulated, and lamin B1 (LMNB1), Annexin A2 (ANXA2), and Annexin A2-like protein (ANXA2P2) were upregulated in low versus high BMD subjects [40]. Of these, ANXA2 has shown increased monocyte transendothelial migration, whereas TAGLN2 is involved in angiogenesis, a process important for bone remodeling, and the rest of the proteins had unknown functions in relation to osteoporosis [40]. On performing Gene Set Enrichment Analysis (GSEA), it was found that platelet activation and growth as well as hemostasis were the most significantly enriched gene sets in low BMD condition, while Weighted Gene Coexpression Network Analysis (WGCNA) revealed that Annexin family genes including ANXA1, ANXA2, ANXA5, and ANXA6 were upregulated in low BMD condition [40]. Further, on overlapping the results from WGCNA and DAVID, it was observed that three Gene Ontology terms were significantly enriched, namely, lipid binding, phospholipid binding, and calcium dependent phospholipid binding [40]. These investigators also analyzed protein-protein interactions using STRING software wherein network associations for Annexin and ubiquitin protein families were predicted [40]. Another recent study on monocyte proteins in relation to BMD in premenopausal Caucasians showed that 30 proteins were differentially expressed, of which ITGA2B, GSN, and RHOA were differentially regulated in the low versus high BMD groups [41]. Network analyses using STRING revealed the enrichment of two pathways: regulation of actin cytoskeleton and leukocyte transendothelial migration [41].

In our study, we used iTRAQ based quantitative proteomics approach to study monocytes from premenopausal and postmenopausal Indian women with low versus high BMD, all in a single experimental platform using 4-plex iTRAQ coupled to nano-LC-MS/MS [42]. We found that heat shock protein 27, also known as heat shock protein beta-1 (HSPB1), was distinctly upregulated in low BMD condition in both premenopausal and postmenopausal categories [42]. Validation revealed that total HSP27 (tHSP27) and phosphorylated HSP27 (pHSP27) were elevated in low BMD condition in both categories not only in monocytes but also in sera [42, 43]. Interestingly, pHSP27 exhibited a higher odds ratio than tHSP27 to predict low BMD, irrespective of menopausal status [43]. Performance characteristics exhibited by pHSP27 and collagen type 1 cross-linked C-telopeptide (CTX-1) were comparable; however, pHSP27 showed lower sensitivity than CTX-1 and lower or comparable specificity than CTX-1 [43]. Besides its well-known antiapoptotic properties, phosphorylated HSP27 increased monocyte migration towards both MCP-1 and RANKL [42]. Further, exogenous phosphorylated HSP27 increased monocyte adhesion onto endothelial cells and monocyte transendothelial migration (unpublished data).

## 5. Monocyte MicroRNA Profiling in Low versus High BMD Condition

MicroRNAs or miRNAs (small noncoding RNAs which are 19–25 nucleotides in length) have emerged as potential biomarkers as they regulate gene expression usually by modulating mRNA stability or translation into protein. Recently, two studies have been reported on miRNA profiling in monocytes in relation to BMD in postmenopausal Caucasian women [44, 45]. In these studies, upregulation of miR-133a [44] and miR-422a [45] was observed in low BMD condition, both of which were confirmed by qRT-PCR. These miRNAs showed negative correlation with the mRNA levels of their target genes, albeit not significant [44, 45]. The elevated levels of miR-133a were also confirmed in serum samples, though in a separate study in postmenopausal Chinese women [45].

## 6. Conclusions and Future Perspectives

Based on “omics” data which has been experimentally validated, molecules in circulating monocytes that are relevant to BMD may be categorized into processes such as apoptosis/survival (PLK3 and HSPB1), migration/chemotaxis (CXCL3, GSN, and HSPB1), adhesion (RSU1, HSPB1), transendothelial migration (ANXA2, HSPB1), differentiation into osteoclasts (STAT1, GBP1, ANXA2, and GSN), and other miscellaneous processes such as histamine biosynthesis (HDC), glucocorticoid sensitivity (GCR), regulation of oxidative stress (SOD2, GPX1), and protein folding (P4HB) and multifunctional molecules, miR-133a and miR422a* (complete list of molecules identified and validated in Table 1)*. Of these, HSPB1 and miR133a have also been found to be elevated in serum levels, pointing towards their importance. However, there are ethnic discrepancies in the differential expression of the molecules involved. So far, only Chinese, Caucasian, and Indian ethnic groups have been explored for altered monocyte gene expression patterns related to BMD. In addition, menopausal status is a key factor which differentiates the underlying pathophysiology of low BMD in premenopausal versus postmenopausal women. A broad understanding of early stages of osteoclastogenesis may be achieved through a meta-analysis of monocyte proteome patterns in low versus high BMD conditions in various ethnicities worldwide. Such studies will not only build our knowledge on the initial stages of osteoclastogenesis including monocyte lineage commitment towards osteoclast but also open up avenues for new additional biomarkers and/or drug targets to improve patient management for PMO.

## Figures and Tables

**Table 1:** List of differentially expressed biomolecules in low versus high bone mineral density (BMD) condition identified using “omics” methods and validated by “qRT-PCR” or using “antibodies.”

Gene name (protein name)	Up- (↑) or down- (↓) regulation in low BMD	Biomolecule/omics method	Ethnicity/average age or age range/menopausal status	Functions relevant to osteoclastogenesis	Author, year
GCR (glucocorticoid receptor)	↑	mRNA/Affymetrix HGU133A GeneChip® array	Caucasian/range: 47–55 years/pre- & postmenopause	Response to steroid hormone stimulus, regulating the expression of STAT1	Liu et al., 2005

HDC (histidine decarboxylase)	↑	mRNA/Affymetrix HGU133A GeneChip array	Caucasian/range: 47–55 years/pre- & postmenopause	Histamine biosynthetic process	Liu et al., 2005

CXCL3 (chemokine (C-X-C motif) ligand 3)	↑	mRNA/Affymetrix HGU133A GeneChip array	Caucasian/range: 47–55 years/pre- & postmenopause	Inflammatory response, prochemotactic	Liu et al., 2005

SOD2 (manganese containing superoxide dismutase)	↑	Protein/2-dimensional electrophoresis	Chinese/range: 20–45 years/premenopause	Removal of superoxide radicals by production of hydrogen peroxide	Deng et al., 2008

GPX1 (glutathione peroxidase 1)	↓	Protein/2-dimensional electrophoresis	Chinese/range: 20–45 years/premenopause	Cellular hydrogen peroxide detoxification	Deng et al., 2008

P4HB (prolyl 4-hydroxylase b subunit)	↓	Protein/2-dimensional electrophoresis	Chinese/range: 20–45 years/premenopause	Protein folding	Deng et al., 2008

RSU1 (Ras suppressor protein 1)	↑	Protein/2-dimensional electrophoresis	Chinese/range: 20–45 years/premenopause	Promotes cell-substrate adhesion	Deng et al., 2008

GSN (gelsolin)	↑ ↓	Protein/2-dimensional electrophoresis	Chinese/range: 20–45 years/premenopause	Actin polymerization and promigratory, podosome assembly	Deng et al., 2008
		Protein/LC-nano-ESI-MS	Caucasian/average 51 years/premenopause	Coregulator of androgen receptor	Deng et al., 2011

STAT1 (signal transducer and activator of transcription 1)	↑	mRNA/Affymetrix HG-U133 plus 2.0 GeneChip array	Chinese/range: 20–45 years/premenopause	Response to interferons, antiviral defence	Lei et al., 2009

GBP1 (guanylate binding protein 1)	↑	mRNA/Affymetrix HG-U133 plus 2.0 GeneChip array	Chinese/range: 20–45 years/premenopause	Antiviral defence, immunity	Lei et al., 2009

ANXA2 (Annexin A2)	↑	Protein/LC-nano-ESI-MS	Caucasian/average 68.2 years/postmenopause	Angiogenesis, transendothelial migration, osteoclast formation	Deng et al., 2011

miR-133a (microRNA 133a)	↑	Small noncoding RNA/ABI TaqMan® miRNA array	Caucasian/average age: 62.6 years/postmenopause	Target genes such as CXCL11 and SLC39A1 inhibit osteoclastogenesis, and CXCR3 expression reduces from monocyte to osteoclast differentiation	Wang et al., 2012

miR-422a (microRNA 422a)	↑	Small noncoding RNA/ABI TaqMan miRNA array	Caucasian/average: 62.6 years/postmenopause	Target genes such as CBL, CD226, PAG1, and TOB2 inhibit osteoclastogenesis, while IGF-1 couples the process of bone remodeling	Cao et al., 2014

HSPB1 (heat shock protein beta-1)	↑	Protein/4-plex iTRAQ LC-MS/MS	Indian/range: 30–40 & 50–60 years/pre- & postmenopause	Stress response, antiapoptotic, promigratory, proadhesive	Daswani et al., 2015

PLK3 (polo-like kinase 3)	↓	mRNA/Affymetrix 1.0 ST arrays	Caucasian/range: 47–56 years/pre- and postmenopause	Proapoptotic, cell cycle checkpoints, DNA damage	Liu et al., 2015

BMD: bone mineral density. LC-MS/MS: liquid chromatography-tandem mass spectrometry. iTRAQ: isobaric tags for relative and absolute quantification.
